# Implementation of an Attention Mechanism Model for Facial Beauty Assessment Using Transfer Learning

**DOI:** 10.3390/diagnostics13071291

**Published:** 2023-03-29

**Authors:** Chao-Tung Yang, Yu-Chieh Wang, Lun-Jou Lo, Wen-Chung Chiang, Shih-Ku Kuang, Hsiu-Hsia Lin

**Affiliations:** 1 Department of Computer Science, Tunghai University, Taichung City 407224, Taiwan; ctyang@thu.edu.tw (C.-T.Y.);; 2 Research Center for Smart Sustainable Circular Economy, Tunghai University, No. 1727, Sec. 4, Taiwan Boulevard, Taichung City 407224, Taiwan; 3 Department of Plastic and Reconstructive Surgery, Craniofacial Research Center, Chang Gung Memorial Hospital, No. 123, Dinghu Rd., Guishan Township, Taoyuan City 333423, Taiwan; 4 Department of Tourism and Recreation Management, Hsiuping University of Science and Technology, No. 11, Gongye Rd., Dali District, Taichung City 412406, Taiwan; 5 Imaging Laboratory, Craniofacial Research Center, Chang Gung Memorial Hospital, No. 123, Dinghu Rd., Guishan Township, Taoyuan City 333423, Taiwan

**Keywords:** transfer learning, deep learning, facial attractiveness prediction, attention mechanism, visualization

## Abstract

An important consideration in medical plastic surgery is the evaluation of the patient’s facial symmetry. However, because facial attractiveness is a slightly individualized cognitive experience, it is difficult to determine face attractiveness manually. This study aimed to train a model for assessing facial attractiveness using transfer learning while also using the fine-grained image model to separate similar images by first learning features. In this case, the system can make assessments based on the input of facial photos. Thus, doctors can quickly and objectively treat patients’ scoring and save time for scoring. The transfer learning was combined with CNN, Xception, and attention mechanism models for training, using the SCUT-FBP5500 dataset for pre-training and freezing the weights as the transfer learning model. Then, we trained the Chang Gung Memorial Hospital Taiwan dataset to train the model based on transfer learning. The evaluation uses the mean absolute error percentage (MAPE) value. The root mean square error (RMSE) value is used as the basis for experimental adjustment and the quantitative standard for the model’s predictive. The best model can obtain 0.50 in RMSE and 18.5% average error in MAPE. A web page was developed to infer the deep learning model to visualize the predictive model.

## 1. Introduction

Facial symmetry in medical plastic surgery has always been an essential factor affecting overall facial attractiveness and youthfulness. Several people have congenital bone problems or temporomandibular joint dysfunction that lead to malocclusion. Not only is the face not conventionally beautiful, but it even affects sleep, difficulty in opening the mouth, pain in chewing, and dysphonia [1,2,3,4].

However, it is challenging to evaluate facial attractiveness manually because it is a somewhat subjective cognitive experience. As such, suppose that a score of zero to five is used as the criterion for assessing the facial attractiveness of different people. In that case, the difference in scores from zero to five may appear for the same face. These differences are due to the distinct aesthetics of each person and the specific social, cultural, and historical context. However, different people still have similar standards when assessing the level of facial attractiveness [5,6]. For example, the ancient Greeks believed that the golden ratio represented beauty’s essence. Leonardo da Vinci’s painting “Mona Lisa” also illustrates that the golden ratio of facial beauty has a particular influence on facial attractiveness.

Fine-grained image classification is a very challenging sub-field in the field of image classification [7,8], because, in the problem of fine-grained image classification, the differences between different classes are often only focused on a small area. In this case, facial attractiveness might be based on the nose’s proportion in the face area or the thickness of the lips. It affects the overall beauty of the face. Therefore, this research aims to use the fine-grained image model to distinguish similar images by first learning features and to use transfer learning to train a model for evaluating facial attractiveness [9,10].

This paper aimed to use fine-grained image classification technology to build a model to evaluate facial attractiveness. In addition to creating a prediction model, this research performs image preprocessing on the dataset. It visualizes the web page’s prediction results. The objectives of this work are as follows:

1. This research uses a fine-grained image model and an attention mechanism model to process the attractiveness assessment of facial images and predict face attractiveness.
2. Compare the impact of different image preprocessing methods on model learning and analyze the difference in model training effectiveness to find the most suitable preprocessing method.
3. Compare the prediction effects of different fine-grained image classification models on facial attractiveness and import the attention mechanism to observe whether the model can effectively improve the accuracy of the model.
4. Visualize the facial attractiveness prediction scores on the webpage, which is convenient for doctors to watch and provides a tool to save the preparation time before surgery.

## 2. Methods

### 2.1. Facial Attractiveness Dataset

Two datasets were used in this study. The first dataset comes from the facial images of patients scanned by 3D imaging in Chang Gung Memorial Hospital from 2016 to 2019. Fifteen doctors attracted 210 facial images. Each pictured face contains fifteen scores.

We averaged all the scores and calculated each picture’s standard deviation based on each picture’s score. Then, based on normal distribution statistics, we initially excluded unqualified pictures based on three times the standard deviation and calculated the average and standard of the score. This is to check whether the scores of the fifteen doctors have too many deviations, so the pictures and scores have no reference value.

**Figure 1:**
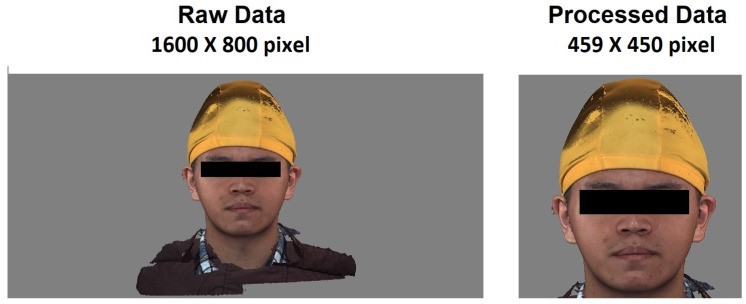
Original picture and processed picture.

Since most of the original pictures are 1600×800 pixels in size, most of the areas in the pictures are gray or black backgrounds. Suppose that these pictures are directly input to the model training. In that case, it will greatly affect the learning effect of the model and add a lot of unnecessary data. To calculate the amount, first use the program to uniformly cut the background on both sides to retain the middle face, and then manually adjust to retain the least part of the background. According to the face size in the picture, most manually adjusted picture sizes are the same. It falls between 400×400 and 500×500. A second screening will make the model more accurate when manually adjusting the image size. For example, in a picture with a high attractiveness score, if there is a blur or a broken image, manually delete the image or fill in the gaps in the middle of the image with color patches of the same color. At the same time, the model learned to manually delete some pictures with blurred scores in different score intervals so that the characteristics between different scores can be more obvious, and there were a total of 189 pictures after the final screening. Figure 1 is a schematic diagram of the original picture and the processed picture.

Another dataset used in this research is called SCUT-FBP5500, which was proposed by the Human–Computer Intelligent Interaction Laboratory of the South China University of Technology. It is a diversified benchmark dataset for facial beauty prediction. The dataset has a total of 5500 sheets. Frontal face images of 350×350 pixels. These images have different attributes, such as men and women from different parts of the world, and of different age groups. The 5500 image dataset can be divided into four types. A subset of different races and genders, including 2000 Asian men (AM), 2000 Asian women (AF), 750 European and American men (CM), and 750 European and American women (CF). These 5500 pictures will be randomly shown to 60 people aged 18–27, and the score range is the lowest from 1 to the highest, and 10% of the pictures will appear twice. A second score will be required if the correlation between the two scoring results is less than 0.7. The 3-fold scoring ensures that the score has a reference value, and finally, the average score of all the picture scores is calculated as the final attractiveness score of this picture.

Before conducting experimental research, we first need to analyze the data. When the machine is performing feature learning, it completely relies on the existing dataset information to learn, so the choice of the dataset is very important. The detail of two distribution scores and the number of data are in different sections of the dataset.

a. SCUT-FBP5500 distribution score:

- From 1 to 2 points—265;
- From 2 to 3 points—3011;
- From 3 to 4 points—1582;
- From 4 to 5 points—642.

b. Chang Gung Hospital distribution score:

- From 1 to 2 points—70;
- From 2 to 3 points—101;
- From 3 to 4 points—18;
- From 4 to 5 points—0.

**Figure 2:**
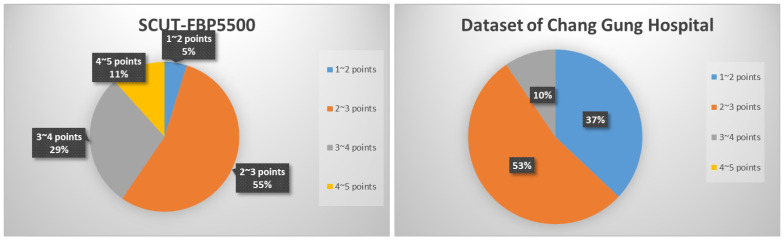
Percentage of data score distribution.

From Figure 2, it can be seen that the proportion of the two datasets has more than 50% for 2–3 points. While the SCUT-FBP dataset has 11% of the data in the interval 4–5, provided in Chang Gung Memorial Hospital. The Chang Gung dataset does not have data with the same score. Therefore, we need to manually downgrade the image data with a score higher than 4. This process aims to avoid low-level data transfer to the data in the Chang Gung dataset after the initial training of the model is completed and the weights are frozen. The same issues also happened for the score range of 1–2 points. The SCUT-FBP dataset only accounts for 5%, but Chang Gung Memorial Hospital has 37%. Therefore, we also need to manually downgrade it to avoid the same problems in the transfer model training.

Since the image dataset and its corresponding scores are on different files, if the Python program is to merge the two and input them into the model for reading training, it will take a lot of time to merge the DataFrame. Thus, manually, the picture numbers and their corresponding scores are unified in a txt file.

### 2.2. Transfer Learning

Transfer learning is an effective technique in computer vision since it makes it possible to quickly construct precise models [11]. Transfer learning begins with the learned patterns when solving a different problem, instead of starting the learning process from scratch. Doing so can make use of prior knowledge and prevent having to begin from scratch. Transfer learning is typically demonstrated in computer vision by using trained models. A model that has already been trained on a sizable benchmark dataset to address a problem resembling the one we are trying to solve is known as a pre-trained model.

Transfer learning is a machine learning method that transfers the knowledge of the original domain to a new domain [12]. As such, the domain can achieve good learning results, and we can divide the data used for migration learning into two categories: one is the source data, and the other is the target data. Source data refer to other data and are not directly related to the task to be solved, while target data are data which are directly related to the task. According to whether the purpose of the two samples is the same, the work of transfer learning is divided into transfer learning, direct push transfer learning, and unsupervised transfer learning [13,14]. According to the technology used in the transfer learning method, the transfer learning method can be roughly divided into (1) transfer learning based on feature selection; (2) transfer learning based on feature mapping; and (3) weight-based transfer learning.

### 2.3. Attention Mechanism

The concept of attention mechanism was first proposed in the field of computer vision in the 1990s. The main inspiration comes from the fact that many different objects may appear simultaneously in the human field of vision, but people’s eyes will be more focused on their interests. Areas or important areas automatically blur the unimportant areas to reduce attention to the rest of the area and focus the limited attention on key information, thereby saving resources and quickly obtaining the most effective information [15]. However, like deep learning, its popularity is limited by hardware computing power and other reasons and gradually fell silent. Until 2014, the Google Mind team used the attention mechanism on the RNN model for image classification [16]. Subsequently, the attention mechanism was widely used in natural language processing tasks based on neural network models such as RNN/CNN. In 2017, Google published Self-Attention, which has also become an important part of the NLP research field. In 2018, Google again proposed two language models, BERT and GPT, making the attention mechanism a popular research avenue. The attention mechanism can be divided into categories according to the calculated area, structure, and model.

The calculation area of attention can be divided into the three following types:

- Soft Attention: This is a relatively common attention method. The weighted probability of all keys is calculated. Each key has a corresponding weight. It is a global calculation method which can also be called Global Attention. This method refers to the content of all keys and then weights them, but the amount of calculation may increase further.
- Hard Attention: This method directly locates a certain key, and the other keys are not considered. The probability equivalent to this key is 1, and the remaining keys are all 0, or part of all keys is selected for use in a set selection method.
- Local Attention: This method is a compromise between the two above methods. An area is calculated first, and the Hard Attention method is used to locate a certain point. With this point as the center, a window area can be obtained in this small area. Then, the soft method is used to calculate attention.

They use different models:

1. CNN + Attention: The convolution operation of CNN can extract important features, but the convolution perception field of CNN is partial, and it is necessary to expand the field of view by superimposing multi-layer convolution areas. In addition, max pooling directly extracts the feature with the largest value, similarly to the hard attention method, and directly selects a feature. There are three ways to use attention on CNN: (1) Attention is performed before the convolutional layer, and attention is performed on the two input sequence vectors at the same time, and the feature vector is calculated, and then spliced into the original vector as the input of the convolutional layer. (2) Perform attention after the convolutional layer as the input to the pooling layer. (3) Perform attention on the pooling layer instead of max pooling.
2. LSTM + Attention: LSTM usually needs to obtain a vector and then make predictions based on the problem. (1) Directly using the last hidden state, this method may lose some shallow information, making it difficult for the model to understand the full text. (2) Perform a weighted average of the hidden state under all steps. (3) Attention mechanism weights the hidden state of all steps, and focuses on the text’s more important hidden state messages. The performance of this method is better than the previous two, and it is convenient to observe which steps are important visually. Still, it is more prone to overfitting and increases the amount of calculation, so additional attention is required.

### 2.4. Fine-Grained Image Classification

The problem of the fine-grained image classification refers to the finer classification of sub-fields in a larger field. Compared with general image classification, the difficulty is that the difference between each sub-category may only be in some smaller features [17]. The common appeal of general image classification is to separate the two domains of cats and dogs that contain more species. It is obvious that regardless of the appearance, outline, color, texture, etc., good resolution results can simply be achieved through several layers of CNN models, while the classification task of fine-grained images is to distinguish the large category of dogs and then make more detailed sub-categories. The following figure is an example. General image classification only needs to distinguish cats. The difference with dogs is that the red frame differs from the blue frame, and the fine-grained picture classification distinguishes Shiba Inu and Akita Inu. More different categories must be subdivided from the red frame because of the different subtypes of the same category of species. Classes often differ only in subtle areas such as ear shape and coat color. We must be able to pay more attention to these tiny features to distinguish them. This type of problem is not only for the classification of pictures but for ordinary people’s eye discrimination ability; if it is not an expert in the field, the problem of fine-grained image classification is also difficult and challenging.

The current training methods for fine-grained image classification can be mainly divided into the following categories:

- Deep convolutional neural network (DCNN) performs fine-grained classification, but this model is ineffective for learning more detailed features, so it is not commonly used.
- The method of using positioning and then identifying: first find the part with the resolution, and then perform feature extraction and classification. This method can be divided into two types: strong supervision and weak supervision.
  1. Strongly supervised learning: add more bounding-box marking information to the network for strongly supervised learning, so that the model can learn the target’s regional information (regional information). However, this method has two disadvantages: (1) It requires a lot of human resources to label the picture; and (2) the manually marked information is not necessarily accurate, but it is the area where the target needs attention.
  2. Weakly supervised learning: Weakly supervised learning is not unsupervised learning, but according to the basic image classification network, only the image category needs to be given. The neural network automatically learns the location of the discriminating area through the model. Then, it pays special attention to the feature difference of this area to identify the target category. Commonly used methods include: image classification based on the attention (attention) mechanism, and the location of the discriminative region is obtained by analyzing the most prominent part of the feature map, such as RA-CNN.
- Integrate different network models: Use multiple deep convolutional neural networks to discriminate similar features in fine-grained recognition, such as the bilinear CNN model used in this study.

### 2.5. Related Works

Before conducting this research, we read the research results in the fields related to the research topic, including theoretical foundations, experimental ideas, implementation processes, etc. These results greatly helped in enabling us to have clearer concepts, avoid many difficulties, and smoothly obtain better research results smoothly.

There have been many studies on facial attractiveness since the 1990s. At first, some standards were formulated based on the ratio of facial features, such as the golden ratio (1:0618) [18], the facial rule of thirds [19], and the new golden ratio [20], and so on. The aforementioned standard ratios evaluate facial attractiveness by using the correlation between the various parts of the face. According to the research of Holland et al., [21], the golden ratio can be applied to evaluating plastic surgery. A universal standard for strength. According to past research, [22,23], it is feasible to use machine learning technology to analyze facial attractiveness based on facial features using standard ratio rules to train models to evaluate facial attractiveness. The experimental results show a significant correlation between the estimated score of the model trained on the standard scale and the score of human subjects. However, since the machine learning model first needs to label the data, it will require higher labor and time costs. Rothe et al. [24] built a convolutional neural network model to learn facial attractiveness from thousands of pictures and applied it to facial beauty recommendations. According to the experimental results, the deep learning model can learn more features that help evaluate facial attractiveness than manually labeled data and features. Sunitha et al. [25] distinguished and classified ethnicity based on facial photos. The feature extractor in the proposed models is an exception network. The feature reduction approach uses the principal component analysis (PCA) technique to overcome the “curse of dimensionality” because the retrieved features are high-dimensional. Additionally, the process of classifying people by their ethnicity is carried out using an ideal kernel extreme learning machine (KELM), with the KELM model’s parameters being tuned using the glow worm swarm optimization (GSO) method.

According to the research of Jinsheng Ji [26] and others, they proposed a new multi-level attention model (MLA-CNN) to classify fine-grained images, and first select feature maps of three sizes through the visual attention mechanism. After entering the model for identification and making final predictions from different feature maps, the experimental results are better than those of other methods in the three challenging fine-grained classification datasets. In the publication of Ting Sun et al. [27], a fine classification system based on CNN was proposed by extracting and interpreting the hierarchical hidden layer features learned by CNN. When evaluating the Caltech-UCSD Birds-200-2011, FGVC-Aircraft car, and Stanford dog datasets, this method uses only labels for training, and no other labeling information can be used for training and testing, and it reaches 83.6% accuracy. In the publication by Qiule Sun et al. [28], they proposed an optimized architecture of a bilinear CNN model. Bilinear CNN performs outer product combinations through the output of the convolutional layer of two CNN models. Nonetheless, generally, bilinear CNN cannot use the inherent information in different convolutional layers, so they proposed a super-layer bilinear pooling CNN (HLBP). In the final test, the accuracy rate can be increased from 88.6% to 91.4%. According to the above conclusions, there are more references for us to choose models from in the experiment.

According to the publication by Zhang et al. [29,30], the degree of facial symmetry is judged through transfer learning, the contour map of the face is used as training data, and the system can be constructed to classify and score the symmetry of the face. Through the transfer of the Xception model, the classification can achieve an accuracy of 90.6%. In the publication of Niu et al. [31], they proposed a new end-to-end fine-grained image classification structure. They added an attention shift mechanism (AS-DNN), automatically locating distinguishable regions and iteratively encoding semantic relevance. According to experimental results, AS-DNN outperforms CNN-based weakly supervised or strongly supervised FGVC algorithms on multiple fine-grained datasets, thus obtaining the best results. Through the visualization of the attention area, this method can locate the area in a complex background. Through the above papers, we need to pay attention to and discuss the use of transfer learning and the transfer of weights and provide references for constructing our system and webpage.

Based on recent research, we found that the use of transfer learning is limited to enhancing the performance of the models in terms of facial recognition models. Therefore, we experimented with the transfer learning methods and fine-grained image classification on our model.

### 2.6. System Architecture

**Figure 3:**
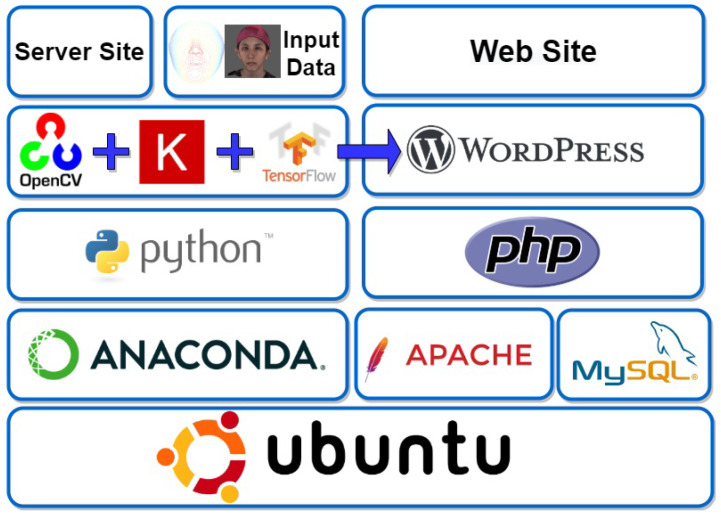
System architecture diagram.

The system in this paper was built on Ubuntu 18.04 system. There are two parts of the system environment. The first part was used for training the models. Face images were preprocessed through OpenCV and Keras. Tensorflow was used for training to generate deep learning models. Different neural networks were used to construct various machine-learning models for experiments. First, using the SCUT-FBP5500 dataset, the model was trained and froze the weights as the pre-trained model. Then, we used the facial images provided by the doctor to train the model using transfer learning from the pre-trained model. After that, model evaluation and performance comparison were performed to select the most suitable model. The second part was the user interface for presenting the prediction based on the selected model. The generated data will be stored in the database through MySQL and presented on the web page through PHP and WordPress frameworks. Figure 3 shows the complete system architecture diagram.

**Figure 4:**
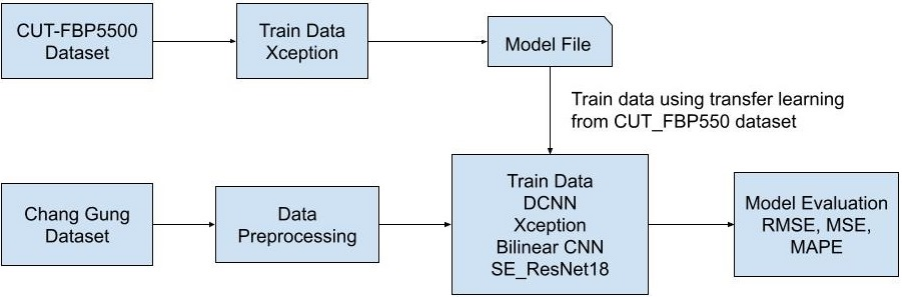
Workflows diagram.

The workflows diagram is depicted in Figure 4.

### 2.7. Data Preprocessing

Every picture must contain noise, but only the difference in severity, such as the noise heard by traditional radio walkie-talkies and radios; or the black and white flickering snowflakes seen on old TVs, are all interfered with by noise. In the field of image processing, noise can be understood as a random change in gray value caused by one or a combination of multiple reasons, such as the current angle of light and shadow, intensity, etc., and the noise may not be directly visible to the naked eye. To distinguish from the picture, picture filtering technology will need to be used at this time. The filter is usually a square matrix with odd sides called mask or kernel. Similarly to the main concept and convolution operation, the filter is calculated with each picture pixel. The filter can be divided into two types according to different application functions:

- Smoothing filter: used to blur and remove noise, including a low-pass filter and Gaussian filter.
- Sharpening filter: strengthen the edge position of the object, including the high pass filter (high pass filter).

**Figure 5:**
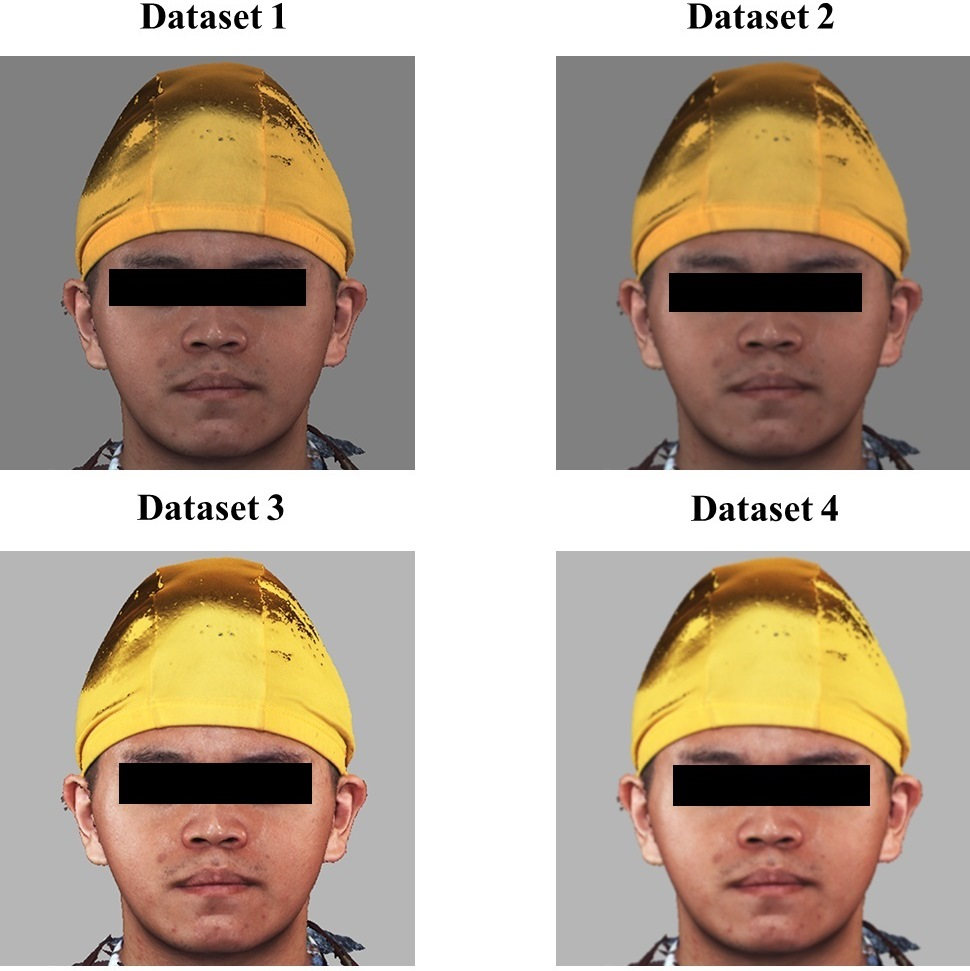
Schematic diagram of the four datasets.

In this experiment, in addition to the use of filters for preprocessing, according to Zhang et al.’s experiment, the brightness and contrast of the images of the Chang Gung Memorial Hospital were adjusted. Using the original dataset for direct training may lead to model learning because the features are not clear enough. Poor or over-fitting, so we will make four different datasets for transfer training and test the model’s performance. The first is without any filtering, only the cropped face images, the second one is the picture processed by the Gaussian filter, the third is the picture after brightness and contrast enhancement, and the fourth is a combination of the second and third methods. Gaussian filtering is performed before brightness and contrast enhancement, as can be seen in dataset in Figure 5.

### 2.8. Training Parameters

**Figure 6:**
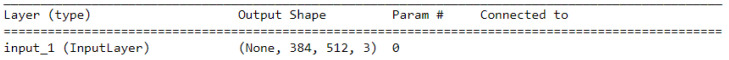
The output shape.

The model was compiled with stochastic gradient descent (SGD) optimizer and early stopping methods. The input and output shapes are described in Figure 6 as follows.

Table 1 shows the training parameters.

### 2.9. Transfer Learning with Pre-Trained Model

This study will use four different models for experiments. The first model is a general sequential DCNN model. A total of five sets of convolutional neural layers with BatchNormalization are established, and a dropout layer is set to 0.25, followed by 3 layers. The fully connected layer has a ReLu start function. The remaining models are the Xception model introduced in the previous section, the bilinear CNN combining VGG16 and ResNet50, and the SE_ResNet18 model with the addition of the SE module. Then, the optimizer and loss function are set. These choices are not arbitrarily selected and need to be adjusted according to the type of neural network and the dataset to be used. The parameter number of each model is as follows.

- DCNN: 30326337;
- Xception: 24961065;
- Bilinear: 37505729;
- SE_ResNet18: 13099777.

1. Xception is an improvement upon Inception v3 proposed by Google [32]. It mainly serves to replace the original Inception v3 convolution layer with depth-wise separable convolution without increasing network complexity, thereby improving the accuracy and speed of the model. We use fine-tuning to adjust the model through the transfer learning method, retain the original learning layer of the model to extract shallow features, and add its data for classification or prediction. The method is to adjust the number of model layers and parameters continuously. Increase the dense layer and the dropout layer to make the model learn better and converge quickly to achieve accurate results. Transfer learning and fine-tuning solve the problem of overfitting in complex neural networks such as Xception when the amount of data is small.
2. Lin et al. proposed a bilinear convolutional neural network model in a paper published in ICCV in 2015 [33]. This paper proposes a better method for obtaining deep convolutional features. This method uses two VGG networks as the reference model. Without using the bounding box to mark the information in the picture, the classification accuracy of 84.1% is reached on the CUB200-2011 dataset. When using bounding, the accuracy rate can be improved by one percent, and the classification accuracy is as high as 85.1%. The bilinear CNN model can effectively identify fine-grained images through a simpler network model.
  On the one hand, the CNN network can achieve the high-level semantic feature acquisition of fine-grained images, and iteratively train the convolution parameters in the network model to filter irrelevant background information in the image. More importantly, on the other hand, model A and model B play a complementary role in the image recognition task, that is, model A can locate the object in the picture, and model B can complete the localization of the object located by model A and perform feature extraction. In this way, the two models can cooperate to complete the class detection and target feature extraction of the input fine-grained image, and better complete the fine-grained image recognition task.
3. The full name of SENet is Squeeze-and-Excitation Networks. It was published by the autonomous driving company Momenta in 2017 and won the championship of the ImageNet image classification that year. It also reduced the best score from 2.991% to 2.251%. It attracted everyone’s attention [34], and SE block is not a complete network model but refers to the unit structure in the network, like the multi-branch structure in the inception model and the residual structure in the ResNet model. The core idea of SENet is to learn feature weights based on the loss value through the network, so that the effective feature map has a larger weight, and the invalid or less effective feature map will reduce the weight. As such, the model can be trained to achieve better results. The SE block embedded in some of the original classification networks inevitably increases some parameters and the calculation time, but with higher accuracy, and the increased calculation performance and space are acceptable.

### 2.10. Evaluation Model

After completing the construction and training of the machine learning model, we need to evaluate the model to understand its effectiveness. There are many ways to evaluate the model. The simple split method mentioned in the previous section is one of them. It is an evaluation method, but only knowing the predicted and real values cannot intuitively reach an understanding of how the model performs. Therefore, we need to have several model evaluation indicators for comparison.

In the second chapter, we mentioned some model evaluation indicators. This study uses RMSE as the loss function when training the model. This will calculate the error value of the model’s training results at each step and assist in learning the model. In the final evaluation of the model, we chose to use MAPE, MAE, and RMSE as the indicators for model evaluation in this study, and use the above three indicators to evaluate the predictive effect of the model.

MAPE is one of the most commonly used evaluation indicators. It can be seen from the following formula that the average absolute percentage error (MAPE) is an error obtained by subtracting the actual value ($\hat{Yi}$) from the predicted value (Yi). Then, the error value is divided by the actual value, so when the actual value is low, and the error is large, it will greatly impact the value of MAPE. The following is the formula of MAPE:

(1) $$MAPE=\frac{100\%}{n}\sum_{i=1}^{n}∥\frac{\hat{Yi}-Yi}{Yi}∥$$

Mean absolute error (MAE) is a commonly used predictive evaluation index, but it has a disadvantage in that it does not consider the average of the actual value. Although an evaluation value can be obtained after calculation, there is no way to know the model. The pros and cons can only be compared by comparison. The following is the formula of MAE:

(2) $$MAE=\frac{1}{n}\sum_{i=1}^{n}∥\hat{Yi}-Yi∥$$

The root mean square error (RMSE) measures the error between the observed and true values. The calculation method does not consider the value of the actual value. Therefore, as long as there is a large error in the prediction result, the value of RMSE will be very poor, and the following formula of RMSE is:

(3) $$RMSE=\sqrt{\frac{1}{n}\sum_{i=1}^{n}{\left(\hat{Y}i-Yi\right)}^{2}}$$

The mean square error (MSE) is the most common indicator in regression problems because it is calculated faster. It measures the average of the squared difference between the predicted value and the actual value. The following is the formula for MSE:

(4) $$MSE=\frac{1}{n}\sum_{i=1}^{n}{\left(\hat{Y}i-Yi\right)}^{2}$$

where *n* = number of times the summation iteration happens and $\hat{Y}i$ = actual value Yi = forecast value.

## 3. Results

### 3.1. Data Preprocessing Experiment

Since the quality of the picture will affect the accuracy of the face score prediction model, it is necessary to test which method is more effective for model learning in the preprocessing of the picture. If the wrong data are selected and input into the model, the predictions will make it difficult for the machine to grasp the relationship between the data, resulting in the poor learning of this model, and inaccurate prediction results. Therefore, to avoid such problems as much as possible, we experiment herein, and we first use three different filters to process the SCUT-FBP5500 dataset and then put it into four models for training, comparing the learning effects of different models in different preprocessing methods, and find the most effective one based on its evaluation indicators—the preprocessing method, and the three filters used, namely the Gaussian filter, median filter, and bilateral filter.

Table 2, Table 3, Table 4 and Table 5 below show the model evaluation indicators for different datasets. According to the training classification of the dataset processed by different filters, MAPE, RMSE, and MAE are used as the evaluation methods. The filter is calculated in one of the four models. The average value of the loss index is used as the basis for judging the pros and cons.

Finally, according to the average result of the evaluation value in Table 6, we can see that the average error value of the Gaussian filter in each indicator is the smallest. Therefore, we think that the Gaussian filter is more suitable for this research than other filters. For the image preprocessing method and in subsequent experiments, we will also use a Gaussian filter for image preprocessing steps.

### 3.2. Initial Weight Training

In this research experiment, we selected four neural networks for training: DCNN, Xception, bilinear CNN, and SEResNet. First, we need to use the SCUT-FBP5500 dataset to train each model to obtain the weight. The training data will serve as the original dataset, and be divided into five subsets using K (K = 5)-fold verification. Each subset will have 1100 images, which will be combined for training and verification. Each model uses the average loss function as a performance evaluation indicator. The training epochs are set to 100, and both the Earlystopping mechanism and the ModelCheckpoint mechanism are set. Earlystopping is set to stop training in advance when the loss value of the model exceeds 10 epochs and does not drop. ModelCheckpoint is in the process of model training. Tensorflow will monitor the loss function of each round of epoch and save the model weight of the epoch with the lowest loss value in this complete training. We used RMSE as the loss function when evaluating the model, which increases the performance of the MAE and MAPE comprehensive consideration model.

The loss value of each model was trained using the SCUT-FBP5500 dataset as a visual presentation. The best loss value of DCNN on the training set is 0.5889, the best loss value of Xception on the training set is 0.2818, and the best loss value of bilinear CNN on the training set is 0.1308, whilst the best loss value of SEResNet on the training set is 0.238. According to the performance ranking on the training set, the bilinear CNN has the best performance, but the performance on the validation set is the three types of DCNN. The numerical performance of the evaluation index is the best, and the experimental results are shown in Table 7.

### 3.3. Transfer Learning

Before the migration training of each model, we need to determine the number of weight layers that each model needs to freeze. In terms of the convolutional neural network architecture, the neural layer closer to the input layer has a larger range of features to learn. The deeper the neural layer, the more localized the learned features, and thus we will freeze 20%, 40%, 60%, and 80% of the neural layer weights in order. We test the freezing of a part of the convolutional layer and retrain the remaining convolutional layer and the fully connected layer, and freeze all the convolutional neural layers and retrain the fully connected layer. We also test whether freezing all convolutional neural layers and part of the fully connected layers and retraining the remaining fully connected layers will have different effects on each model, because having more frozen neural layers means that fewer neural layers need to be trained. Therefore, it is necessary to gradually reduce the learning rate with the percentage of the frozen neural layer to avoid an excessively high learning rate affecting the weight update too fast and the model cannot learn the best effect.

Then, the dataset provided by Chang Gung Memorial Hospital and the frozen weights are used for transfer learning. Since the number of images in the Chang Gung Memorial dataset is small, if we use K-fold to split multiple sub-datasets, the model learning may be unbalanced due to imbalance in the dataset. There is a gradient explosion or overfitting. Therefore, for cutting small datasets, we used the holdout cross validation method to select 80% of the 198 images from each score interval as the training set and 20% as the validation set. After the selection is completed, the post-training set has 146, and the verification set is forty-three. The model will establish the same model structure as in the initial training, and read in the saved weights. Depending on the model structure, the final output layer will be frozen or more neural layers will need to be frozen.

#### 3.3.1. DCNN Freezing Weight Experiment

When performing experiments, each model uses the same dataset to perform freezing weight experiments, and each time a different degree of the neural layer is frozen, five experiments are performed. Furthermore, the average value of these five evaluation indicators is calculated and compared with other experimental results. The best percentage will be used as the number of frozen weight layers for the transfer learning of this model. Table 8 below shows the experimental results of the DCNN model. The field 20% means freezing 20% of the weights close to the input layer, retraining the remaining 80% of the remaining neural layer, and so on. The table results show that the more layers of the DCNN model are frozen, the better the learning effect. It also shows that the migration weight is a significant help for the DCNN model learning, and subsequent experiments will be performed at 80% freezing.

#### 3.3.2. Xception Freezing Weight Experiment

Table 9 is the Xception model experimental results. The field 20% means to freeze 20% of the weights close to the input layer, retrain the remaining 80% of the remaining neural layer, and so on. The table results show that freezing more layers of the Xception model has no effect on the learning, which also shows that the transfer weight does not significantly help the Xception model learning.

#### 3.3.3. Bilinear CNN Freezing Weight Experiment

Table 10 shows the experimental results of the bilinear CNN model. The field 20% means to freeze 20% of the weights close to the input layer, retrain the remaining 80% of the remaining neural layer, and so on. The table results show that the more layers of the bilinear CNN model are frozen, the slightly more the learning effect is improved. It also shows that the migration weight is helpful for learning the bilinear CNN model, and subsequent experiments will be performed at 80% freezing.

#### 3.3.4. SEResNet Freezing Weight Experiment

Table 11 is the experimental result of the SEResNet model. The field 20% means to freeze 20% of the weights close to the input layer, retrain the remaining 80% of the remaining neural layer, and so on. From the table results, it can be seen that freezing more layers of the SEResNet model means that the learning effect will be improved, but freezing too many layers will slightly decrease the learning effect, which also shows that the transfer weight is helpful for SEResNet model learning. In subsequent experiments, the freeze will be 60%.

We can calculate the average loss of each model in Table 12. We can judge that the bilinear CNN model has the best average performance among the three indicators and the prediction results of the four models. Datasets that only use Gaussian filters also have the lowest prediction differences. In the three models except for the Xception model, having more frozen layers indicates greater gradual improvement in the model effect.

Based on the above inferences, we believe that, in this experiment, using a Gaussian filter as the preprocessing method, using a bilinear CNN model, and freezing 80% of the weight as a deep learning prediction model can result in the best performance and the lowest average error.

## 4. Conclusions and Future Work

This research compares three filter processing techniques: Gaussian, median, and bilateral. After applying different filters to the same dataset, the dataset is put into the neural network model for training. Then, which preprocessing technology is more suitable for this study is compared through different evaluation indicators. The choice of the smallest average index is the more suitable preprocessing method, which can provide a better picture dataset of the learning model to provide a better learning effect. In the part of the experimental data processing of this study, we carried out different cutting methods according to the size of the dataset and used the K-fold splitting method of K = 5 on the SCUT-FBP5500 dataset to divide the dataset into five subsets. Each subset is 1100 pictures, which are used as training and validation data. In model training, training and validation data can be used to confirm the learning situation of the model. After model training, evaluation, and testing, we can better understand the prediction effect of this prediction model to facilitate subsequent modification and optimization. In this study, four types of neural networks were chosen to build deep learning models, including the sequential deep convolutional neural network model (DCNN), the convolutional network model Xception with branch structure design, and the other two models. The neural network models of the attention mechanism are the ResNet18 neural network model and the SE module, i.e., the bilinear convolutional neural network model. These two neural network models focus on different parts of the image for feature extraction, effectively evaluate the facial attractiveness scores, and use MAPE as a model evaluation method to compare the prediction accuracy and error values of different neural network prediction models. Finally, we create a web page and system through WordPress to provide users with a convenient and fast-use environment. As long as the user uploads a face image, preprocessing and call model prediction through the back-end server can quickly predict the results presented on the front-end web page. In the future, this research will continue to test more different attention mechanism models, use other regression analysis methods for prediction, and then add more different datasets for facial attractiveness for pre-training, because the currently used dataset, SCUT-FBP5500, is mainly based on Asian faces. Westerners such as in Europe and the United States have less facial data, and due to the influence of different cultures, social customs, and aesthetics, they will have different effects on the ratings of attractiveness, so it is expected that if more scores from different people can be added, the accuracy of the prediction model will be improved. After the prediction model is improved, a complete face attractiveness evaluation webpage is hoped to be established, and the data will be more comprehensively visualized here. On the platform, it is provided as a reference for users in need.

## Figures and Tables

**Table 1:** Training Parameters.

Training Methods	Parameters
**SGD**	lr = 0.0001
	decay = $1\times 10^{-6}$
	momentum = 0.9
	nesterov = True
**Early Stopping**	monitor = ‘val_loss’
	patience = 10
	verbose = 0
	mode = ‘min’
**Training**	batch size = 8
	epoch = 30
	verbose = 1

**Table 2:** Evaluation table for the prediction results of the original dataset.

Evaluation Indicators	DCNN	Xception	SEResNet	Bilinear CNN
RMSE	1.09883	0.897755	0.86641	0.927477
MAE	0.890495	0.714845	0.686824	0.742291
MAPE	27.0327	24.7454	23.0226	26.1041

**Table 3:** Evaluation table for the prediction results of the Gaussian dataset.

Evaluation Indicators	DCNN	Xception	SEResNet	Bilinear CNN
RMSE	0.8543	0.8993	0.898461	0.9166
MAE	0.652	0.717	0.711041	0.73622
MAPE	20.25	24.96	23.1653	26.2611

**Table 4:** Evaluation table for the prediction results of the median dataset.

Evaluation Indicators	DCNN	Xception	SEResNet	Bilinear CNN
RMSE	1.10167	0.894011	0.975473	0.873147
MAE	0.896717	0.711264	0.778013	0.699094
MAPE	27.1277	24.3483	24.8788	24.6128

**Table 5:** Evaluation table for the prediction results of the bilateral dataset.

Evaluation Indicators	DCNN	Xception	SEResNet	Bilinear CNN
RMSE	0.887057	0.903688	0.913588	0.930038
MAE	0.680141	0.719672	0.722979	0.740271
MAPE	20.8158	24.8288	23.438	24.5602

**Table 6:** Average loss value of evaluation index.

Evaluation Indicators	Original	Gaussian	Median	Bilateral
RMSE	0.947618	0.892166	0.961075	0.908593
MAE	0.758614	0.704065	0.771272	0.715766
MAPE	25.2262	23.0591	25.2419	23.4107

**Table 7:** Model prediction result table.

	DCNN	Xception	SEResNet	Bilinear CNN
RMSE	0.844	0.892	0.89	0.881
MAE	0.643	0.709	0.71	0.701
MAPE	19.90	24.392	25.01	24.39

**Table 8:** DCNN model weight experimental result table.

	20%	40%	60%	80%
RMSE	4.305	4.12	2.873	1.553
MAE	4.199	4.008	2.701	1.276
MAPE	94.98	90.4	59.43	26.04

**Table 9:** Xception model weight experimental result table.

	20%	40%	60%	80%
RMSE	1.22	1.218	1.207	1.372
MAE	0.977	0.978	0.974	1.097
MAPE	22.45	22.48	22.698	23.315

**Table 10:** Bilinear CNN model weight experimental result table.

	20%	40%	60%	80%
RMSE	1.107	1.144	1.386	1.158
MAE	0.908	0.938	1.109	0.923
MAPE	23.33	26.671	22.729	19.467

**Table 11:** SEResNet model weight experimental result table.

	20%	40%	60%	80%
RMSE	2.307	1.044	1.008	1.04
MAE	2.1	0.849	0.8043	0.8249
MAPE	45.225	19.919	19.04	19.22

**Table 12:** Model prediction average loss.

	DCNN	Xception	Bilinear CNN	SEResNet
RMSE	0.5295	1.197	0.505	0.512
MAE	0.4277	0.98	0.41	0.412
MAPE	18.5	22.312	18.5	19.17

## Data Availability

The data presented in this article are not readily available because of ethical reasons. Requests to access the data should be directed to the corresponding authors.
